# Environmental sustainability in eye-care services

**DOI:** 10.22336/rjo.2023.19

**Published:** 2023

**Authors:** Consuela-Mădălina Gheorghe

**Affiliations:** * Philologist, Authorized translator

Environmental sustainability is a concept that has been increasingly discussed in the last decade, since scientists are trying to generate evidence to assess the impact of eye-care services on the environment and to decrease the rates of eye diseases. Due to demographic changes and the development of eye-care services in low-and middle-income countries, the expansion of eye services provision needs to be environmentally sustainable (Buchan JC, Thiel CL, Steyn A, Somner J, Venkatesh R, Burton MJ, Ramke J. Addressing the environmental sustainability of eye health-care delivery: a scoping review. Lancet Planet Health. 2022 Jun; 6(6):e524-e534. doi: 10.1016/S2542-5196(22)00074-2.).

To preserve environmental sustainability in eye-care services, a few simple steps should be taken: people should buy and use plastic free ophthalmological products, should make sure that their ophthalmological purchases have environmental benefits, should pay attention to the labels of the ophthalmological products, should try to recycle the ophthalmological products and substances (if the products and substances can be recycled), etc. 

Thus, like in other fields, in Ophthalmology, environmental sustainability is defined by the 7 Rs: rethink, refuse, reduce, reuse, repair, regift, recycle (https://www.aeromatico.com/the-7-rs-of-sustainability/). Rethinking refers to the fact that people should ask themselves if they really need to buy certain ophthalmological products as consumer choice can help drive ophthalmological product companies to adjust their packaging. Refusing means not utilizing single-use plastic products or non-recyclable packaging of the ophthalmological products. Reducing deals with rationalizing the consumption and making informed decisions regarding the ophthalmological products. Reusing means that ophthalmological items can be reused even if they are old, as long as they are still usable (not broken or expired), such as eyeglasses frames, but not contact lenses, because they have an expiration period and they should not be used by more than one person (because of hygienical and eye health issues). Moreover, the eyeglasses frames or sunglasses without diopters can also be repaired instead of buying new ones, or they can be regifted if not necessarily needed by the person who already bought them. Last, but not least, recycling is one of the most important actions, which can be taken if the person decides to throw away the ophthalmological product, due to different reasons. In such a case, people should benefit from recycling programs and centers available, close to their areas, or even have a designated recycle bin. An example of recycling program is the one undergone for some time by many of the optician shops, which sell not only lenses but also frames. What the customers have to do is bring their old eyeglasses frames to the designated shops and obtain a discount when buying a new frame or benefit from the services offered by that certain shop. 

Sustainability in eye care is very important, especially in the local eye clinics, which are the only providers of eye care for a very large geographical area. Therefore, it is of utmost importance that the eye clinics retain their ability to operate autonomously through a sustainable business model.

At the same time, one of the factors of the success of any eye-care clinic is financial sustainability. This can be accomplished by having eye services that any consumer can afford to pay for. Unfortunately, not all consumers afford the eye-care services. 

Strengthening the eye-care sustainability implies various dimensions such as leadership, core services, human resources, management, technology, etc. 

It is said that acknowledging the existence of a climate emergency represents the first step in the commitment of an organization to act first regarding environmental sustainability. Thus, actions can be taken to reduce carbon emissions and prioritize initiatives. 

Human resources individuals can help by raising the awareness and need of climate change, thus influencing the others to act on changing their practices. 

Sustainability can be achieved by reducing the carbon footprint, this meaning that ophthalmological equipment, technology and consumables should have a lower environmental impact and organizations should adhere more and more to this. 

Moreover, the energy consumption in ophthalmological facilities should be reduced by using a more efficient building design and even solar energy. 

In conclusion, to be a sustainable clinical practice, four principles should be followed (https://www.eyenews.uk.com/features/ophthalmology/post/sustainability-in-eyecare-climate-action-in-eyecare):

1. Ophthalmological disease prevention and health promotion;

2. Patient education and empowerment;

3. Angular eye health service delivery;

4. Utilization of ophthalmological medical procedures and technologies with a lower impact on the environment. 

